# Potential Biomarkers in Diagnosis of Renal Acanthamoebiasis

**DOI:** 10.3390/ijms22126583

**Published:** 2021-06-19

**Authors:** Karolina Kot, Patrycja Kupnicka, Oliwia Witulska, Aleksandra Czepan, Natalia Agnieszka Łanocha-Arendarczyk, Aleksandra Anna Łanocha, Danuta Izabela Kosik-Bogacka

**Affiliations:** 1 Department of Biology and Medical Parasitology, Pomeranian Medical University in Szczecin, Powstańców Wielkopolskich 72, 70-111 Szczecin, Poland; kotkar@pum.edu.pl (K.K.); oliwiaewawitulska@gmail.com (O.W.); aleksandra.czepan@wp.pl (A.C.); nlanocha@pum.edu.pl (N.A.Ł.-A.); 2 Department of Biochemistry and Medical Chemistry, Pomeranian Medical University in Szczecin, Powstańców Wielkopolskich 72, 70-111 Szczecin, Poland; pkupnicka@gmail.com; 3 Department of Haematology and Transplantology, Pomeranian Medical University in Szczecin, Unii Lubelskiej 1, 71-252 Szczecin, Poland; aleksandra.lanocha@pum.edu.pl; 4 Independent Laboratory of Pharmaceutical Botany, Pomeranian Medical University in Szczecin, Powstańców Wielkopolskich 72, 70-111 Szczecin, Poland

**Keywords:** *Acanthamoeba* spp., kidney, KIM-1, MCP-1, MMPs, NGAL

## Abstract

Recent studies indicate that *Acanthamoeba* spp. may play a significant role in kidney dysfunction. The aim of the study was to examine the levels of kidney injury molecule 1 (KIM-1), neutrophil gelatinase-associated lipocalin (NGAL), and monocyte chemotactic protein 1 (MCP-1), as well as an activity of matrix metalloproteinases 2 and 9 (MMP-2 and MMP-9, respectively) in the kidneys of immunocompetent and immunosuppressed mice infected with *Acanthamoeba* spp. The levels of KIM-1, NGAL, and MCP-1 were analyzed by enzyme-linked immunosorbent assay (ELISA), and the activity of MMPs was determined by gelatin zymography. The elevated KIM-1 level was found in the kidneys of immunocompetent mice at the beginning of *Acanthamoeba* spp. infection. In the immunosuppressed mice, the KIM-1 level was statistically different. The statistically decreased NGAL level was found in the kidneys of immunocompetent mice compared to the uninfected mice. In the immunocompromised mice, we found statistically significant differences in MCP-1 levels between the uninfected and infected groups. There was an increase in the expression of both MMP-2 and MMP-9 in the kidneys of immunocompetent and immunosuppressed mice infected with *Acanthamoeba* spp. compared to the uninfected mice. The results indicate that KIM-1, NGAL, MCP-1, MMP-2, MMP-9, and MMP-9/NGAL might be promising biomarkers of renal acanthamoebiasis.

## 1. Introduction

*Acanthamoeba* spp. are free-living protozoa with pathogenic properties. The main biotopes of these amoebas are the brain, lungs, and cornea [1]. Recent studies also found the presence of amoebas in the kidneys of hosts with disseminated acanthamoebiasis. However, the presence of *Acanthamoeba* spp. in the kidneys is usually determined *postmortem* as serum and urine biochemistry are not always abnormal, and may vary depending on the immune status of the host and the amoebic strain [2,3,4]. Histological examinations of the kidneys of hosts infected with *Acanthamoeba* spp. showed increased proliferation of cell nuclei in the proximal and distal tubular epithelium, mitotic figures, single lymphocytes [4], bloody ecchymoses, and inflammatory foci [5]. Extensive necrotic lesions of the renal tubules and glomeruli were also found in hosts infected with the environmental strain of *Acanthamoeba* [5]. Additionally, patients infected with *Acanthamoeba* spp. may develop acute kidney injury which seems to be related with secondary infections or the amphotericin B and/or rifampicin, which are used in acanthamoebiasis therapeutic schemes [3]. Therefore, it is important to identify sensitive and early markers indicating changes in the kidneys in disseminated acanthamoebiasis.

Biomarkers that can be used in the diagnosis of kidney injury are kidney injury molecule-1 (KIM-1), neutrophil gelatinase-associated lipocalin (NGAL), matrix metalloproteinases (MMPs), and monocyte chemotactic protein 1 (MCP-1), also known as the chemokine CC motif ligand 2 (CCL2) [6,7,8].

KIM-1 is a type I membrane glycoprotein with a molecular weight of about 104 kDa that is located mainly on the apical surface of the proximal tubule of the nephron in the outer core layer [9,10]. Increased expression and synthesis of KIM-1 occurs only in response to hypoxia or damage to the renal tubules [11,12]. According to Huo et al. [13], KIM-1 has many features of an ideal biomarker of kidney damage: (i) It is detected only in the event of damage to the proximal tubules, (ii) it is possible to measure its concentration in urine non-invasively, and (iii) the concentration of this protein in urine correlates with its expression in the kidneys and the degree of their damage [13]. Studies have shown that KIM-1 may serve as an early marker of kidney dysfunction of various etiologies, including after cardiac surgery, in extracorporeal circulation, kidney transplantation, in chronic renal failure, and in acute ischemic tubular necrosis [14,15,16].

NGAL is an extracellular protein with a molecular weight of 25 kDa [17]. NGAL expression was found in cells of the immune system, prostate, trachea, stomach, large intestine, and renal tubular cells. NGAL mediates the immune response. It is involved in cell apoptosis and nephrogenesis by stimulating the differentiation of mesenchymal cells towards the renal tubular epithelium [18,19]. NGAL undergoes glomerular filtration and reabsorption in the proximal tubules. The increase in its expression and synthesis is the result of damage of the proximal tubules due to the inflammatory process, neoplasms, and ischemia [20]. Urinary and serum NGAL correlates with the degree of renal damage [21]. In the renal tubules, NGAL mRNA is expressed within a few hours after the action of the damaging factor. Therefore, NGAL is considered to be a good renal biomarker involved in the pathophysiological process of acute kidney injury (AKI) [22]. Moreover, NGAL may be an indicator of renal ischemia associated with tubular necrosis, renal failure after renal transplantation, and chronic renal failure [23].

The MCP-1, also known as CCL-2, is the most well-known CC chemotactic chemokine family. MCP-1 is expressed in endothelial cells, fibroblasts, and mononuclear cells. In the kidney, cell types producing MCP-1 are tubular cells, smooth muscle cells, mesangial cells, podocytes, and also infiltrating cells such as eosinophils and mast cells [24,25]. MCP-1 is secreted in response to signals such as proinflammatory cytokines and it plays an important role in controlling the recruitment of leukocytes in inflammation and tissue injury [26,27,28]. MCP-1 contributes to a variety of renal diseases, including AKI, chronic kidney disease (CKD), chronic rejection of renal transplantation, IgA nephropathy, lupus nephritis, and diabetic nephropathy [29,30,31]. MCP-1 is suggested to reflect the level of tubular injury and renal inflammation [32].

MMPs are proteins that play many important roles in biological processes. Their proteolytic activity significantly affects the composition and structure of the extracellular matrix (ECM) through the degradation of its components and the regulation of signal particles. MMPs are involved in the regulation of cells for differentiation, proliferation, apoptosis, adhesion, and migration, ensuring normal renal glomerular and tubular function [33,34,35]. In the kidney, MMPs are expressed in the glomeruli, proximal and distal tubules, and collecting tubules [33,34]. MMPs are associated with both physiological and pathological processes in the kidneys. MMPs are involved in AKI, chronic kidney disease, diabetic nephropathy, lupus nephritis, post-infectious glomerulonephritis, and chronic transplant rejection [36]. It has been suggested that measuring the levels of MMPs in plasma and serum can help in the diagnosis of various kidney diseases, as well as help in monitoring the treatment of patients with renal insufficiency undergoing dialysis or in kidney transplant patients. However, no reference value of MMP has been developed yet that could be a marker of normal kidney function [37].

Many of the new renal biomarkers have been widely studied in the most common renal diseases but have been scarcely investigated in parasitic diseases. Kidney injury identification in parasitosis is often diagnosed only when the disease is fully established, with clear clinical signs and symptoms of renal dysfunction. Renal dysfunction in parasitosis is an important cause of medical complications [38]. The aim of the study was to examine the levels of kidney injury molecule 1 (KIM-1), neutrophil gelatinase-associated lipocalin (NGAL), and monocyte chemotactic protein 1 (MCP-1), as well as an activity of matrix metalloproteinases 2 and 9 (MMP-2 and MMP-9, respectively) in the kidneys of immunocompetent and immunosuppressed mice infected with *Acanthamoeba* spp.

## 2. Results

### 2.1. KIM-1

**Figure 1:**
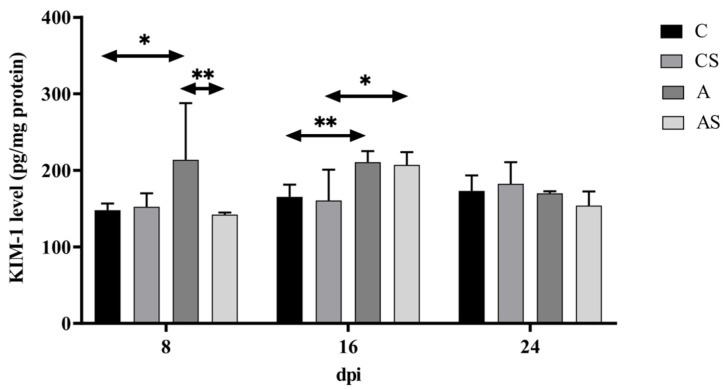
The level of kidney injury molecule-1 (KIM-1) in the kidneys of mice infected with *Acanthamoeba* spp. KIM-1 was determined using ELISA and it is expressed in pg/mg protein. Data represent the means ± standard deviation for six independent experiments. (A: Immunocompetent mice infected with *Acanthamoeba* spp.; AS: Immunosuppressed mice infected with *Acanthamoeba* spp.; dpi: Days post *Acanthamoeba* spp. infection; C: Immunocompetent mice uninfected with *Acanthamoeba* spp.; CS: Immunosuppressed mice uninfected with *Acanthamoeba* spp.; statistically significant differences * *p* < 0.05; ** *p* < 0.01).

In immunocompetent mice infected with *Acanthamoeba* spp., there was a tendency towards downregulation in the level of KIM-1. However, it was not statistically significant. The highest KIM-1 level was found at the beginning of the infection, on the 8th-day post-*Acanthamoeba* spp. infection (dpi). There was a statistically significant higher level of KIM-1 in the group of infected immunocompetent mice at 8 dpi (U = 16, *p* = 0.03) and 16 dpi (U = 0, *p* < 0.01) compared to the uninfected immunocompetent mice (Figure 1).

In immunosuppressed mice, the KIM-1 level decreased at 8 dpi, then increased at 16 dpi, and re-decreased at 24 dpi (H = 16.16, *p* < 0.001). The highest level of KIM-1 was found on the 16th-day post-*Acanthamoeba* spp. infection. A statistically significant higher KIM-1 level was found only in the group of infected immunosuppressed mice at 16 dpi than in uninfected immunosuppressed mice (U = 8, *p* = 0.01; Figure 1).

Taking into account the immune status of the hosts, KIM-1 levels were lower in the immunosuppressed infected group compared to the immunocompetent infected group. A statistically significant difference was found only at 8 dpi (U = 0, *p* < 0.01; Figure 1).

### 2.2. NGAL

**Figure 2:**
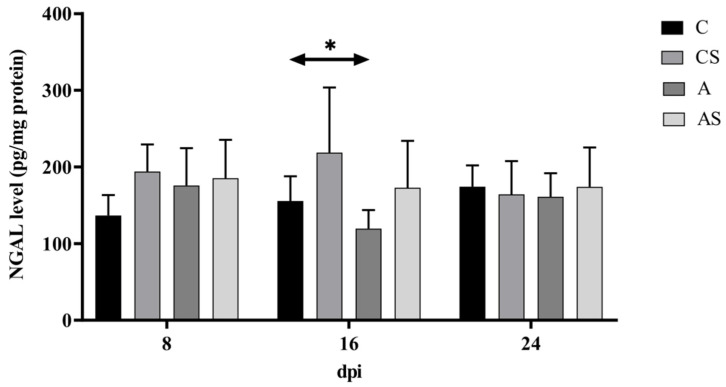
The level of neutrophil gelatinase-associated lipocalin (NGAL) in the kidneys of mice infected with *Acanthamoeba* spp. NGAL was determined using ELISA and it is expressed in pg/mg protein. Data represent the means ± standard deviation for six independent experiments. (A: Immunocompetent mice infected with *Acanthamoeba* spp.; AS: Immunosuppressed mice infected with *Acanthamoeba* spp.; dpi: Days post *Acanthamoeba* spp. infection; C: Immunocompetent mice uninfected with *Acanthamoeba* spp.; CS: Immunosuppressed mice uninfected with *Acanthamoeba* spp.; statistically significant difference * *p* < 0.05).

In immunocompetent mice, the NGAL level increased at 8 dpi, then decreased at 16 dpi, and re-increased at 24 dpi (H = 6.17, *p* < 0.05). The highest NGAL level was found at the beginning of the infection, on the 8th-day post-*Acanthamoeba* spp. infection. However, a statistically significant difference was found between the group of infected and uninfected immunocompetent mice at 16 dpi (U = 8, *p* = 0.02). A lower level of NGAL was observed in *Acanthamoeba* spp.-infected mice (Figure 2).

In immunosuppressed mice infected with *Acanthamoeba* spp. the level of NGAL was similar. The highest level of NGAL was found on the 8th-day post-*Acanthamoeba* spp. infection. No statistically significant differences were found between the group of infected and uninfected immunosuppressed mice (Figure 2).

Taking into account the immune status of the hosts, no statistically significant differences were found.

### 2.3. MCP-1

MCP-1 levels were similar in immunocompetent mice infected with *Acanthamoeba* spp. The highest MCP-1 level was found on the 16th-day post-*Acanthamoeba* spp. infection. There were no statistically significant differences between the group of infected and uninfected immunocompetent mice.

**Figure 3:**
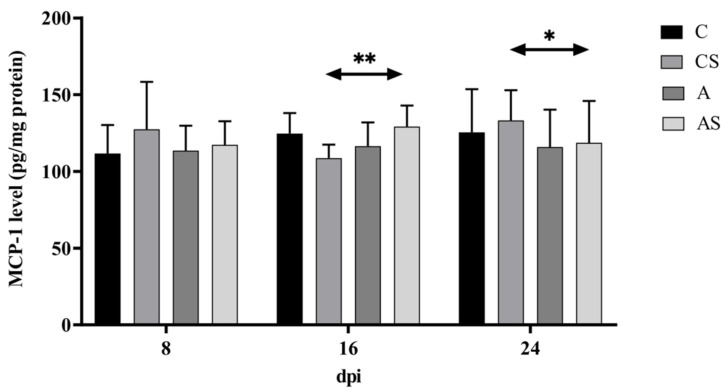
The level of monocyte chemotactic protein 1 (MCP-1) in the kidneys of mice infected with *Acanthamoeba* spp. MCP-1 was determined using ELISA and it is expressed in pg/mg protein. Data represent the means ± standard deviation for six independent experiments. (A: Immunocompetent mice infected with *Acanthamoeba* spp.; AS: Immunosuppressed mice infected with *Acanthamoeba* spp.; dpi: Days post *Acanthamoeba* spp. infection; C: Immunocompetent mice uninfected with *Acanthamoeba* spp.; CS: Immunosuppressed mice uninfected with *Acanthamoeba* spp.; statistically significant differences * *p* < 0.05; ** *p* < 0.01).

In immunosuppressed mice infected with *Acanthamoeba* spp. MCP-1 levels were similar. The highest level of MCP-1 was found on the 16th-day post-*Acanthamoeba* spp. infection. There was a statistically significant higher level of MCP-1 in *Acanthamoeba* spp.-infected mice at 16 dpi (U = 4, *p* < 0.01) and statistically significant lower level in *Acanthamoeba* spp.-infected mice at 24 dpi (U = 20, *p* = 0.02) compared to the uninfected animals (Figure 3).

Taking into account the immune status of the hosts, no statistically significant differences were found.

### 2.4. MMPs

**Figure A1:**
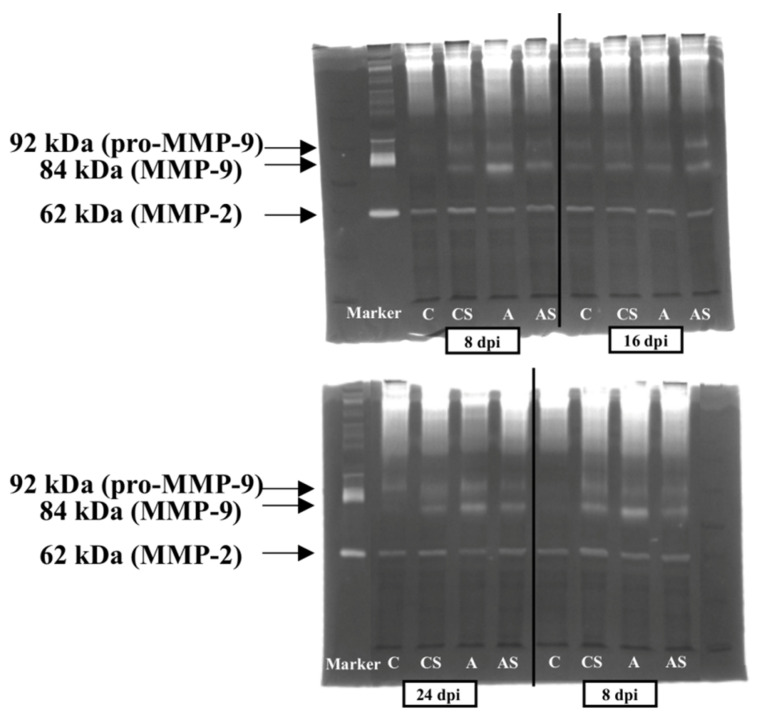
Visualization of gelatin zymography determining the level of matrix metalloproteinase 2 (MMP-2) and 9 (MMP-9) and matrix pro-metalloproteinase (pro-MMP-9) in immunocompetent mice infected with *Acanthamoeba* spp. A: Immunosuppressed mice infected with *Acanthamoeba* spp.; AS: Immunocompetent mice uninfected with *Acanthamoeba* spp.; C: Immunosuppressed mice uninfected with *Acanthamoeba* spp.; CS: At 8, 16, and 24 days post-infection (dpi).

The activity of matrix metalloproteinases for all the studied groups is shown in Figure A1 (Appendix A). There were three bands on the gel that were identified based on their molecular weight: Pro-MMP-9 (92 kDa), MMP-9 (84 kDa), and MMP-2 (62 kDa).

#### 2.4.1. MMP-2

**Figure 4:**
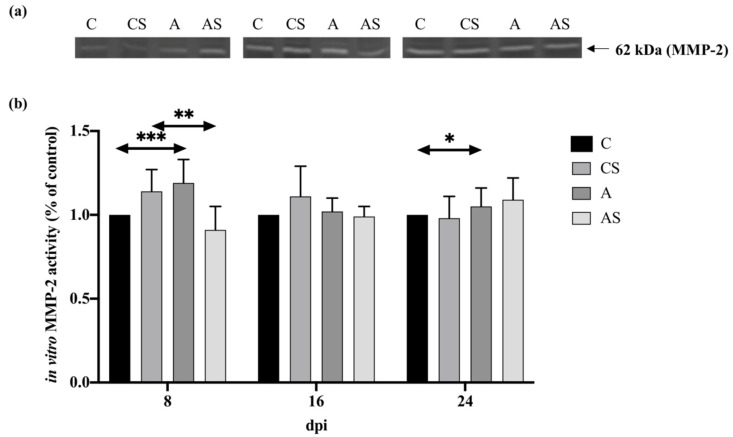
Activity of matrix metalloproteinases-2 (MMP-2) in the kidneys of mice infected with *Acanthamoeba* spp. MMP-2 was determined using zymography. Representative zymography (**a**) and densitometric analysis of MMP-2 protein (**b**) in mouse kidneys were shown. Data represent the means ± standard deviation for six independent experiments. (A: Immunocompetent mice infected with *Acanthamoeba* spp.; AS: Immunosuppressed mice infected with *Acanthamoeba* spp.; dpi: Days post *Acanthamoeba* spp. infection; C: Immunocompetent mice uninfected with *Acanthamoeba* spp.; CS: Immunosuppressed mice uninfected with *Acanthamoeba* spp.; statistically significant differences * *p* < 0.05; ** *p* < 0.01; *** *p* < 0.001).

The MMP-2 activity in the kidneys of immunocompetent mice at 8 dpi increased and then decreased during the *Acanthamoeba* spp. infection. At 16 and 24 dpi, the levels of MMP-2 in the kidneys of immunocompetent mice were similar. There was a statistically significant difference between the control group vs. 8 dpi vs. 16 dpi vs. 24 dpi (H = 21.62, *p* = 0.001). The level of MMP-2 in the kidneys of immunocompetent mice infected with *Acanthamoeba* spp. was higher compared to the control group on each day of the infection. However, statistically significant differences were found between 8 dpi and the control group (U = 0.00, *p* < 0.001) and between 24 dpi and the control group (U = 7.00, *p* < 0.05; Figure 4).

In the kidneys of mice with decreased immune response, the MMP-2 activity increased with the duration of the infection. However, the result between groups was statistically insignificant. There was a lower level of MMP-2 at 8 and 16 dpi compared to the control group and an increased level in 24 dpi compared to the uninfected animals. However, a statistically significant difference was only found between the 8 dpi and the control group (U = 4.00, *p* <0.01; Figure 4).

Comparing the immune status of the animals, a higher activity was noted at 8 and 16 dpi in a group of immunocompetent mice infected with *Acanthamoeba* spp., and at 24 dpi a higher level of MMP-2 was observed in the group of immunosuppressed mice infected with *Acanthamoeba* spp. A statistically significant difference was found only between the group of immunocompetent and immunosuppressed hosts on the 8th-day post-*Acanthamoeba* spp. infection (U = 0.00, *p* < 0.001).

#### 2.4.2. Pro-MMP-9

**Figure 5:**
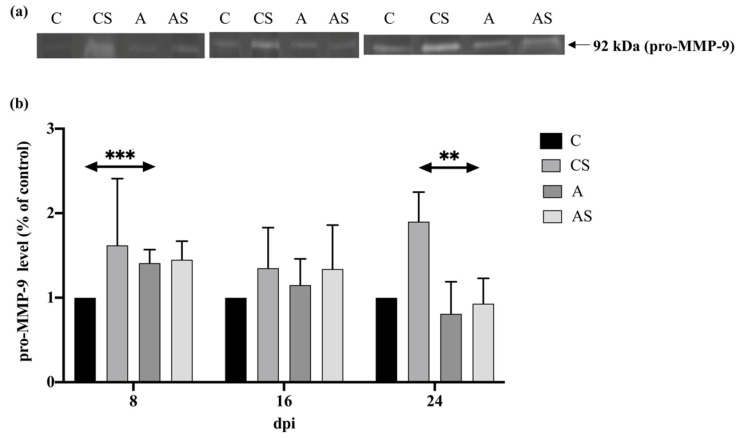
Pro-metalloproteinases-9 (pro-MMP-9) levels in the kidneys of mice infected with *Acanthamoeba* spp. pro-MMP-9 was determined using zymography. Representative zymography (**a**) and densitometric analysis of pro-MMP-9 protein (**b**) in mouse kidneys were shown. Data represent the means ± standard deviation for six independent experiments. (A: Immunocompetent mice infected with *Acanthamoeba* spp.; AS: Immunosuppressed mice infected with *Acanthamoeba* spp.; dpi: Days post *Acanthamoeba* spp. infection; C: Immunocompetent mice uninfected with *Acanthamoeba* spp.; CS: Immunosuppressed mice uninfected with *Acanthamoeba* spp.; statistically significant differences ** *p* < 0.01; *** *p* < 0.001).

In the kidneys of immunocompetent mice infected with *Acanthamoeba* spp., the level of pro-MMP-9 decreased with the duration of the infection (H = 22.96, *p* <0.001). There was a statistically significant difference in the pro-MMP-9 level between 8 dpi and the control group of mice (U = 0, *p* < 0.001; Figure 5).

In the kidneys of immunosuppressed mice infected with *Acanthamoeba* spp., there was also a decrease in pro-MMP-9 levels during the course of the infection. However, it occurred to be statistically insignificant. There was a statistically significant decrease in the level of pro-MMP-9 at 24 dpi compared to the uninfected mice (U = 0, *p* < 0.01; Figure 5).

Comparing the immune status of hosts, no statistically significant differences were found between immunocompetent and immunosuppressed animals.

#### 2.4.3. MMP-9

**Figure 6:**
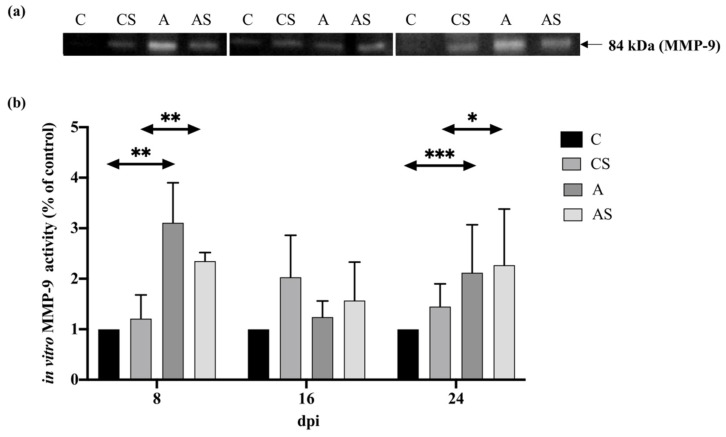
Activity of matrix metalloproteinases-9 (MMP-9) in the kidneys of mice infected with *Acanthamoeba* spp. MMP-9 was determined using zymography. Representative zymography (**a**) and densitometric analysis of MMP-9 protein (**b**) in mouse kidneys were shown. Data represent the means ± standard deviation for six independent experiments. (A: Immunocompetent mice infected with *Acanthamoeba* spp.; AS: Immunosuppressed mice infected with *Acanthamoeba* spp.; dpi: Days post *Acanthamoeba* spp. infection; C: Immunocompetent mice uninfected with *Acanthamoeba* spp.; CS: Immunosuppressed mice uninfected with *Acanthamoeba* spp.; statistically significant differences * *p* < 0.05; ** *p* < 0.01; *** *p* < 0.001).

In the kidneys of immunocompetent mice, an increase in the MMP-9 activity was found at 8 days post-*Acanthamoeba* spp. infection, followed by a decrease at 16 dpi and a re-increase at 24 dpi (H = 32.66, *p* < 0.001). There was a statistically significant difference in the MMP-9 level between mice at 8 dpi and the control group (U = 0.00, *p* < 0.01) and between the 24 dpi and uninfected animals (U = 0.00, *p* < 0.001; Figure 6).

In the kidneys of immunosuppressed mice, an increase in the MMP-9 activity was found at 8 days post-*Acanthamoeba* spp. infection, followed by a decrease at 16 dpi and a re-increase in 24 dpi (H = 8.33, *p* < 0.05). There were increased levels of MMP-2 at 8 and 24 dpi compared to the uninfected mice and a decreased level at 16 dpi compared to the control group. Statistically significant differences were found between dpi and uninfected animals (U = 0.00, *p* < 0.001) and between 24 dpi and the control group (U = 12.00, *p* < 0.05; Figure 6).

Comparing the immune status of the animals, a higher activity was found at 8 dpi in a group of immunocompetent mice infected with *Acanthamoeba* spp. At 16 and 24 dpi, higher levels of MMP-9 were observed in the group of immunosuppressed mice infected with *Acanthamoeba* spp. A statistically significant difference was found only between immunocompetent and immunosuppressed mice at 8 days post-*Acanthamoeba* spp. infection (U = 0.00, *p* < 0.01).

### 2.5. MMP-9/NGAL

In the kidneys of immunocompetent mice infected with *Acanthamoeba* spp., the MMP-9/NGAL ratio decreased with the duration of the infection (H = 25, *p* < 0.001). There was a statistically significant difference in the MMP-9/NGAL ratio between 8 dpi and the control group of mice (U = 5, *p* < 0.05) and between 16 dpi and uninfected animals (U = 5, *p* < 0.01).

In the kidneys of immunosuppressed mice infected with *Acanthamoeba* spp., there was no difference between the control vs. 8 dpi vs. 16 dpi vs. 24 dpi. A statistically significant difference in the MMP-9/NGAL ratio was observed between 8 dpi and uninfected mice (U = 2, *p* < 0.05).

Comparing the immune status of hosts, no statistically significant differences were found between immunocompetent and immunosuppressed hosts.

## 3. Discussion

The most common biomarker of kidney dysfunction is creatinine since it does not bind to plasma proteins, its metabolism extends beyond the kidneys, is freely filtered in the glomeruli, and is not reabsorbed in the renal tubules [39,40]. Unfortunately, there are some limitations to the use of serum creatinine as an ideal biomarker of AKI or CKD. Factors affecting serum creatinine include muscle mass and dietary protein content, therefore creatinine levels are related to age, muscle mass, gender, and race. Currently, the most important problem with the use of creatinine in the diagnosis of kidney disease is the fact that it is a relatively late marker and changes in its level are visible only after about 50% loss of functional kidney function [41]. In parasitic diseases, renal dysfunction is not always reflected in the serum creatinine level of the hosts. In some hosts infected with *Leishmania* spp. and *Toxoplasma gondii*, no differences in creatinine levels were found, despite the fact that both parasites are characterized by high tropism to the kidneys [42,43,44]. In the case of renal acanthamoebiasis, creatinine is also not a sensitive marker of renal dysfunction [45]. Since the level of creatinine as a marker of kidney damage in parasitic diseases is not precise, new, more effective biomarkers are being sought. Early detection of kidney damage will allow for quick implementation of treatment with monitoring of its effectiveness [39]. Currently, two main groups of biomarkers of kidney damage have been identified: tubular and glomerular. The first includes KIM-1, NGAL, MCP-1, and the second includes MMPs [46]. In the studies concerning biomarkers levels in the hosts infected with varied parasites, the proteins were determined in the urine of the hosts. In our study, we examined the proteins in the kidney tissue. However, the urine levels of these biomarkers correlate with their kidney expressions [47,48].

### 3.1. KIM-1

The levels of KIM-1 were examined in hosts infected with *Leishmania* spp. and *Plasmodium* spp. In patients with visceral leishmaniasis (VL), Meneses et al. [49] examined urine KIM-1 (uKIM-1). The mean uKIM-1 level was above 3 times higher in patients with VL and acute kidney injury (AKI group) compared to healthy patients, above 2 times higher in patients with VL and without acute kidney injury (non-AKI group) compared to healthy individuals, and almost 1.5 times higher in AKI group compared to the non-AKI group. However, a statistically significant difference was reported only between the AKI group vs. healthy subjects. Van Wolfswinkel et al. [50] determined KIM-1 in the urine of patients with imported *P. falciparum,* and only one patient showed elevated uKIM-1 levels. Punsawad and Viriyavejakul [51] examined KIM-1 in the kidney tissues from fatal *P. falciparum* cases with and without AKI. The authors reported a higher expression of KIM-1 in the AKI group compared to the non-AKI and control group of patients. Meneses et al. [49], van Wolfswinkel et al. [50], and Punsawad and Viriyavejakul [51] suggested that KIM-1 is not a promising biomarker for early detection of nephropathy in visceral leishmaniasis and malaria. Contrary to the aforementioned studies, we suggest that KIM-1 can be considered for the early detection of renal acanthamoebiasis.

In the present study, the elevated KIM-1 level was found in the kidneys of immunocompetent mice at the beginning of infection (8 dpi) and it lasted to 16 dpi. On the last day of infection, the KIM-1 level decreased and it was at a similar level as in the control group of mice. In the immunosuppressed mice, the KIM-1 level at the beginning of infection was at a similar level as in the uninfected mice. However, at 16 dpi, KIM-1 increased and it was statistically significantly different compared to control animals.

Pianta et al. [52] determined uKIM-1 in rats after the dexamethasone treatment. The uKIM-1 slightly increased after dexamethasone administration but with a delay. The authors, similar to Kabat-Koperska et al. [53], did not find any influence of immunosuppressive treatment on the KIM-1 levels. In our study, immunosuppression did not influence on the KIM-1 levels in the kidneys, since the protein levels were similar in both control groups. However, we suggest that immunosuppression delayed the synthesis of KIM-1 that is visible as an increased level of KIM-1 only at the 16th-day post-*Acanthamoeba* spp. infection in the immunosuppressed mice and the lower KIM-1 level at 8 dpi in the kidneys of immunocompromised infected mice compared to the immunocompetent infected group.

### 3.2. NGAL

The levels of NGAL were examined in hosts infected with *Leishmania* spp. and *Plasmodium* spp. Peris et al. [54] determined NGAL in urine and serum samples (uNGAL and sNGAL, respectively) of dogs experimentally infected with *L. infantum*. There were no significant differences in sNGAL levels, whereas urinary NGAL to the creatinine ratio (uNGAL/C) was statistically significantly different between the control group and proteinuric dogs infected with *L. infantum*, as well as between non-proteinuric dogs infected with *L. infantum* and proteinuric dogs infected with *L. infantum* [54]. Meneses et al. [49] examined sNGAL and uNGAL in patients with VL. The mean sNGAL level was statistically increased in the serum of patients with VL and AKI compared to patients with VL and without AKI, and it was increased in the non-AKI group of patients compared to healthy participants. The uNGAL level was significantly higher only in the AKI group compared to healthy individuals. Van Wolfswinkel et al. [50] determined sNGAL and uNGAL in patients with imported *P. falciparum*. The sNGAL and uNGAL were significantly higher in the AKI group compared to the non-AKI group of patients. Plewes et al. [55] examined urine NGAL corrected urine creatinine (uNGAL/Ucr), and they observed a statistically higher level uNGAL/Ucr in the patients with severe AKI compared to patients with moderate AKI, mild AKI, and without AKI. The authors of the aforementioned studies [49,54] suggested that NGAL can be considered as a good biomarker of leishmaniasis with kidney involvement. It is also suggested that NGAL can be used to detect malaria-induced AKI since NGAL excretion continues with the ongoing renal stress [50,55].

In our study, we also found a statistically significant difference between the control group and 8, 16, and 24 dpi. However, contrary to the authors studying kidney involvement in leishmaniasis and malaria, in the kidneys of immunocompetent mice at 16 dpi, we found a statistically decreased NGAL level compared to the uninfected mice, which may be caused by other factors [56,57]. In the immunosuppressed mice, there were no statistically significant differences in the NGAL level.

### 3.3. MCP-1

The levels of MCP-1 were examined in hosts infected with *Leishmania* spp. and *Plasmodium* spp. Oliveira et al. [42] examined urine MCP-1 (uMCP-1) in patients with VL. The uMCP-1 was significantly elevated in the patients with VL compared to the control group of patients. Oliveira et al. [42] reported that high levels of uMCP-1 in patients with VL had normal serum creatinine levels, suggesting the presence of glomerular inflammation and incipient renal damage. Meneses et al. [49] also examined uMCP-1 in patients with VL. The mean uMCP-1 level was above 5.5 times higher in the patients with VL and AKI compared to healthy individuals, above 3 times higher in the patients with VL but without AKI group compared to the control group, and ~1.75 times higher in the patients with VL and AKI group compared to the patients with VL and without AKI. However, a statistically significant difference was noted only between the AKI group vs. healthy patients. Even though the uMCP-1 involvement in renal inflammation and nephropathy in patients with VL was reported by Oliveira et al. [42], Meneses et al. [49] reported that uMCP-1 was not related to AKI development in patients infected with *Leishmania* spp. Ibarra-Meneses et al. [58] also reported that MCP-1 is a promising robust biomarker for confirming asymptomatic individuals exposed to *Leishmania infantum*. It may also provide a marker of the efficacy of treatment for visceral leishmaniasis. Elias et al. [59] determined renal tissue protein expression of MCP-1 in an experimental model of severe malaria. Statistically elevated MCP-1 levels were observed at 6 and 7 days post-*Plasmodium* spp. infection compared to the control group of mice. The authors suggested that MCP-1 might be a good biomarker of nephropathy in malaria [59].

In the present study, we found no statistically significant differences in MCP-1 levels in the kidneys of immunocompetent mice. In the immunocompromised mice, we found statistically significant differences in MCP-1 levels between the 16 dpi and control group, as well as between 24 dpi and control group. It is important to point out, that MCP-1 levels were statistically different compared to the control groups of mice at those days when the morphological changes in the kidneys were most visible in the histopathological examinations [4].

### 3.4. MMPs

The role of MMPs in the kidney in parasitic infections is not fully understood. To date, the expressions of MMPs have only been studied in the kidney of the host in the course of malaria. Van den Steen et al. [60] examined the expression of all MMPs in the kidneys of *Plasmodium* spp. infected BALB/c mice. The level of MMPs in the kidneys was determined at 2, 4, 6, and 8 dpi. The authors found a statistically significantly lower expression of MMP-2 and MMP-11 on the 6th- and 8th-day post-*Plasmodium* spp. infection, and a lower expression of MMP-15 at 8 dpi. In contrast, the MMP-14 expression was increased in mouse kidneys at 6 and 8 dpi, and the MMP-9 expression was increased at 8 dpi. Punsawad and Viriyavejakul [51] examined only the expression of MMP-3 in autopsied kidneys of *P. falciparum*-infected patients. The MMP-3 expression was higher in the kidneys of AKI patients compared to patients without AKI infected with *P. falciparum* and to patients in the control group. Punsawad and Viriyavejakul [51] suggest that MMP-3 may be activated directly by the mitogen-activated protein kinase (MAPK), by reactive oxygen species (ROS) or by pro-inflammatory cytokines [50,55]. Van den Steen et al. [60] and Punsawad and Viriyavejakul [51] indicate that MMPs may serve as specific markers in the course of AKI caused by *Plasmodium* spp. infection.

In the present study, there was an increase in the activation of MMP-2 and MMP-9 in the kidneys of immunocompetent mice infected with *Acanthamoeba* spp. compared to the control group. A statistically significant increase in activation of MMP-9 in the kidneys of mice with a lower immune status was also found at the beginning of *Acanthamoeba* spp. infection.

Recent reports indicate that measuring the level of MMPs in serum or urine may help in the diagnosis of renal dysfunction [37]. Han et al. [61] found that the presence of MMP-9 in urine may be a biomarker of AKI, and Zhou et al. [62] showed that the presence of MMP-7 in urine may serve as a biomarker of renal fibrosis. However, MMP-2 and MMP-9 also play a role in the process of renal fibrosis [63]. The increased expression of MMP-2 and MMP-9 reported in this study in the kidneys of mice infected with *Acanthamoeba* spp. may also be caused by the process of fibrosis. Along with the progressive process of fibrosis, tubular epithelial cells often show hypoxia, which may lead to an increase in the expression of gelatinases [64]. The histopathological examination of the kidneys of mice infected with *Acanthamoeba* spp. revealed a lighter color of the nucleus and cytoplasm of the renal tubules [4], which may indicate a process of fibrosis. This process is characterized by a lighter color on histological examination. Further analyses, including the examination of the level of TGF-β (cytokine involved in the pathogenesis of fibrosis) in the kidneys of mice infected with *Acanthamoeba* spp., are necessary to confirm or reject the proposed hypothesis.

### 3.5. MMP-9/NGAL

NGAL has the ability to bind with MMP-9. When they exist in complex, there is less degradation of MMP-9 due to the effects on NGAL on the stability of MMP-9. This results in a higher gelatinolytic activity of MMP-9 on the extracellular matrix [65]. Perrin et al. [66] observed that high concentrations of MMP-*9*/NGAL in the serum have been associated with shorter progression-free survival and poor overall survival of renal carcinoma [17]. Korzeniecka-Kozerska et al. [67] reported that the MMP-9/NGAL ratio may serve as a differentiation marker between minimal change nephrotic syndrome and focal segmental glomerulosclerosis in children. The MMP-9/NGAL ratio in the kidneys, urine or serum samples of hosts with parasitosis has not been investigated yet.

In the present study, the MMP-9/NGAL ratio might be considered as a biomarker of immunocompetent hosts infected with *Acanthamoeba* spp., whereas in immunosuppressed mice, the MMP-9/NGAL ratio is a promising marker of early diagnosis of kidney dysfunction since the ratio increased at the beginning of the infection.

### 3.6. Possible Mechanisms in Renal Acanthamoebiasis

**Figure 7:**
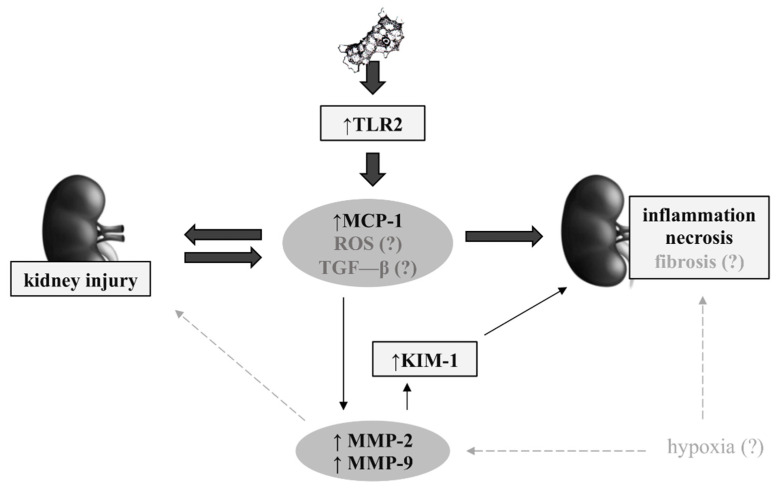
The possible pathways of pathomechanism of renal acanthamoebiasis (KIM-1: Kidney injury molecule 1; MCP-1: Monocyte chemotactic protein 1; MMP-2: Matrix metalloproteinase 2; MMP-9: Matrix metalloproteinase 9; NGAL: Neutrophil gelatinase-associated lipocalin; ROS: Reactive oxygen species; TGF-β: Transforming growth factor-β; TLR2: Toll-like receptor 2). Black bolded arrows - data from the previous studies; black arrows – data from the present study; grey arrows – possible pathways.

Based on the present study and the articles published so far, it is known that *Acanthamoeba* spp. activate TLR2 in the kidneys of mice [4]. Activation of TLR2 increases significantly and enhances the pro-inflammatory response of a tissue, with the participating of numerous cytokines and chemokines, including MCP-1 [68,69]. The TLR may also induce reactive oxygen species production (ROS) and the synthesis of transforming growth factor-β (TGF-β) [70,71]. In the early stage, MCP-1 may induce kidney injury, while prolonged MCP-1 synthesis may lead to inflammation and even necrosis in the renal tubes of mice. Using the same experimental model, Kot et al. [4] observed histopathological changes in the kidneys on those days when MCP-1 was statistically significantly different. Increased expression and activity of MMP-2 and MMP-9 due to the most likely secreted proinflammatory cytokines and/or hypoxia in the kidneys, lead to ECM degradation, which causes increased damage to the basal membrane of the kidneys [63]. MMPs also activate the synthesis of KIM-1 [12]. There are reports on the role of KIM-1 in the process of interstitial renal fibrosis. Renal tubules with a high content of KIM-1 have been shown to be surrounded by inflammatory cells and exhibit fibrotic features, and their glomerulus focal and segmental sclerosis [72]. However, studies of cytokines, oxidative stress parameters, and hypoxia are necessary to confirm this suggestion (Figure 7).

## 4. Materials and Methods

### 4.1. Ethics Statement

Consent from the Local Ethics Committee for Scientific Experiments on Animals in Szczecin (no. 29/2015 of 22 June 2015) and Poznań (no. 64/2016 of 9 September 2016) were obtained to conduct the experiment on laboratory animals. All animal experiments were performed in a strict agreement with good animal practice with the recommendations in the Guide for Care and Use of Laboratory Animals.

### 4.2. Acanthamoeba Spp.

*Acanthamoeba* spp. (AM22) were isolated from the bronchoaspirate fluid of a patient with chronic leukaemia and atypical pneumonia. The AM22 strain was analyzed by molecular methods and T16 genotype was detected [73]. The amoebas were kept on non-nutrient agar (NNA) with deactivated bacteria plates in 37 °C.

### 4.3. Animal Model

BALB/c mice (n = 96), obtained from the Center for Experimental Medicine of the Medical University of Bialystok, were about 6–10 weeks old and had health certificates issued by a veterinarian. The animals were divided into four groups: (i) Immunocompetent mice infected with *Acanthamoeba* spp. (group A; n = 30), (ii) immunosuppressed mice infected with *Acanthamoeba* spp. (group AS; n = 30), (iii) immunocompetent mice uninfected with *Acanthamoeba* spp. (group C; n = 18), (iv) immunosuppressed mice uninfected *Acanthamoeba* spp. (CS group; n = 18). To reduce immunity, animals from the AS and CS groups were administered intraperitoneally with 0.22 mg (10 mg/kg body weight) methylprednisolone (Solu-Medrol, MPS) dissolved in 0.1 mL 0.1% saline solution daily for 4 days prior to infection with *Acanthamoeba* spp. Group A and AS mice were inoculated by intranasal inoculation with 3 μL of a suspension containing 10–20 thousand amoebas. The animals from C and CS groups were administered the same volume of saline (3 μL 0.9% NaCl). Animals were sacrificed at 8, 16, and 24 days post-*Acanthamoeba* spp. infection (dpi) after intraperitoneal administration (i.p.) of sodium pentobarbital (Euthasol vet, FATRO, Raamsdonksveer, the Netherlands; 2 mL/kg body weight). The kidneys were sterile collected from animals using sterile equipment, fixed in liquid nitrogen, and then stored at −80 °C until analyses. More details about the animal model are given by Łanocha-Arendarczyk et al. [74].

### 4.4. Homogenization of Samples

The frozen kidneys of mice were placed in a dewar with liquid nitrogen. The samples were individually and carefully transferred to a metal homogenizer cooled with liquid nitrogen. The tissues were comminuted by hitting a chilled metal mandrel with a hammer. The prepared sample was distributed to new Eppendorf tubes. The samples were stored in a freezer at −80 °C until further testing. After each use, the metal homogenizer was cleaned, rinsed with ethanol, and cooled again in liquid nitrogen.

### 4.5. Determination of Protein Content

Tissue homogenates were treated with a lysis buffer pH 8.5 (50 mM Tris-HCl, 150 mM NaCl, and 0.5% Triton X-100). Samples were mixed thoroughly and incubated on ice for 15 min, followed by centrifugation (20 min, 14,000 rpm, 4 °C), after which the supernatant was removed. The total protein concentration in the supernatant was determined by the BCA method using a commercial MicroBCA Protein Assay Kit (Thermo Scientific, Pierce Biotechnology, Waltham, MA, USA) according to the manufacturer’s instructions. The absorbances of the samples were determined spectrophotometrically using an EZ Read 2000 plate reader from Biochrom (wavelength 562 nm). The protein concentration was determined using a standard curve prepared simultaneously with the use of solutions with the known protein (albumin) concentration.

### 4.6. KIM-1

KIM-1 levels were assessed using the Quantikine ELISA Mouse TIM-1/KIM-1/HAVCR immunoassay according to the manufacturer’s instructions (cat. no. MKM100; R&D Systems, Inc., Minneapolis, MN, USA). The buffer with the samples, control, and standards were added to 96-well plates and incubated at room temperature for 2 h. Then, after washing a cold mouse KIM-1 conjugate was added and the plates were incubated for 1 h at 2–8 °C. After second washing, the substrate solution was added to each well and incubated for 30 min at room temperature in the dark. Following the treatment with a stop solution, the plates were read at 450 nm using a microplate reader (EZ Read 2000, Biochrom Ltd., Cambridge, UK).

### 4.7. NGAL

NGAL was assessed using the Mouse Lipocalin-2 ELISA Kit according to the manufacturer’s instructions (cat. no. ELM-Lipocalin-2; RayBiotech, Norcross, GA, USA). This assay used an antibody specific for mouse Lipocalin-2 coated on a 96-well plate. Standards and samples were added into the wells and the Lipocalin-2 present in a sample is bound to the wells by the immobilized antibody. The incubation lasted 2.5 h at room temperature. The wells were washed and the biotinylated anti-mouse Lipocalin-2 antibody was added. The incubation lasted 1 h at room temperature. Then, after washing the streptavidin solution was added and the plates were incubated for 45 min at room temperature. After washing away the unbound antibody, the HRP-conjugated streptavidin was added to the wells. The wells were washed again, and a TMB substrate solution was added to the wells. After 30 min, the stop solution was pipetted and the plates were read at 450 nm using a microplate reader (EZ Read 2000, Biochrom Ltd., Cambridge, UK).

### 4.8. MCP-1

MCP-1 was assessed using the Mouse MCP-1 ELISA Kit according to the manufacturer’s instructions (cat. no. BMS6005; Invitrogen USA, Carlsbad, CA, USA). An anti-mouse MCP-1 coating antibody was adsorbed onto the microwells. Each sample, standard, and control sample were added to the plates and a mouse MCP-1 presented in the sample or standard binded to the antibodies was adsorbed to the microwells. A biotin-conjugated anti-mouse MCP-1 antibody was added and binds to mouse MCP-1 captured by the first antibody. The incubation lasted 2 h at room temperature. Following incubation, the unbound biotin-conjugated anti-mouse MCP-1 antibody was removed during a wash step. Streptavidin-HRP is added and binds to the biotin-conjugated anti-mouse MCP-1 antibody. The incubation lasted 1 h at room temperature. Following incubation, the unbound Streptavidin-HRP was removed during a wash step, and the substrate solution reactive with HRP was added to the wells. The incubation lasted 10 min at room temperature. Following the treatment with the stop solution, the plates were read at 450 nm using a microplate reader (EZ Read 2000, Biochrom Ltd., Cambridge, UK).

### 4.9. MMPs

The separation of proteins was done in a polyacrylamide gel consisting of the separating (bottom) and thickening (top) layer.

In order to perform the MMP-2 and MMP-9 activity analyses, gels with 7.5% acrylamide content and 1% gelatin content were prepared. The protein concentration of each sample was calculated so that each sample contained 30 µg of protein. The samples were diluted 4-fold with a Laemmli Buffer (Laemmli Sample Buffer, Bio-Rad, Feldkirchen, Germany). The final sample volume was 15 µl. The samples were loaded onto the prepared gel which was placed in an electrophoretic chamber filled with an electrode buffer (25 mM Tris/250 mM glycine/0.1% SDS; Merck, Germany). A mass standard was applied to the gel (a mixture of 10 recombinant proteins (10–250 kDa); Precision Plus Protein ™ All Blue Prestained Protein Standards, BioRad, Hercules, CA, USA), a standard containing the tested metalloproteinases (Bovine Serum Albumin, Biowest, France) and test samples. The electrophoresis was carried out under the voltage: 125 V for 90 min at the temperature of 4 °C. After electrophoresis was completed, the gel was incubated in a renaturation buffer (2.5% Triton X-100, Thermo Fisher Scientific, Waltham, MA, USA) at room temperature to elute with SDS and restore the enzymatic activity after the separation of proteins in the gel. After the renaturation buffer was removed, the gel was rinsed with distilled water and then incubated in an incubation buffer containing the cofactors necessary for the gelatin hydrolysis reaction with the studied MMPs (50 mM Tris, 10 mM CaCl2, 0.02% NaN3, pH = 7.4, Sigma-Aldrich, Germany). Incubation was carried out at 37 °C for 16 h. The gel was stained with a Coomassie blue solution (Coomassie Brilliant Blue R-250, Thermo Fisher Scientific, Waltham, MA, USA). The gel was then washed several times with a decolorizing solution (10% methanol, 5% acetic acid; Merck, Germany) until distinct bands were obtained. Pictures of gels were taken using a transilluminator (Molecular Imager ChemiDock XRS +, Bio-Rad, Hercules, CA, USA). The densitometric analysis was performed using the Image Lab Software 6.1.0 (Bio-Rad Laboratories, Inc., Hercules, CA, USA).

### 4.10. Statistical Analysis

Statistical analysis was performed using StatSoft Statistica 12.0 and Microsoft Excel 2016. The arithmetic mean (AM) and standard deviation of the arithmetic mean (SD) were calculated. The non-parametric U-Mann Whitney and Kruskal-Wallis test was used to assess the differences between the studied parameters. Significant statistical differences were assumed when *p* < 0.05.

## 5. Conclusions

Our study suggests that KIM-1, MMP-2, MMP-9, and MMP-9/NGAL can be promising biomarkers in the diagnosis of renal acanthamoebiasis in immunocompetent hosts. KIM-1, MMP-2, and MMP-9 might be a biomarker of early detection of kidney involvement in *Acanthamoeba* spp. infection in immunocompetent hosts. In immunosuppressed hosts, KIM-1, MCP-1, MMP-9, and MMP-9/NGAL can be promising biomarkers in the diagnosis of renal acanthamoebiasis, wherein MMP-9 and MMP-9/NGAL might be a biomarker of early detection.

In future research, it is important to check the studied biomarkers in the urine and/or serum of hosts infected with *Acanthamoeba* spp. to make the diagnosis of renal acanthamoebiasis as quick and minimally invasive as possible. Moreover, due to the fact that the studied biomarkers may be present in infections or diseases caused by other agents than amoebas, the research for potential and ideal markers of renal acanthamoebiasis should be continued.

## Data Availability

Derived data supporting the findings of this study are available from the corresponding author (D.K.-B.) on request.
